# Prevalence of people with sickle cell disease and leg ulcers in Brazil: Socioeconomic and clinical overview

**DOI:** 10.1371/journal.pone.0274254

**Published:** 2022-09-09

**Authors:** Josimare Aparecida Otoni Spira, Eline Lima Borges, Antônio Carlos Martins Guedes, Paula Gabriela Ribeiro Andrade, Vera Lúcia de Araújo Nogueira Lima

**Affiliations:** 1 Departamento de Enfermagem Básica, Escola de Enfermagem, Universidade Federal de Minas Gerais, Belo Horizonte, Minas Gerais, Brazil; 2 Departamento de Clínica Médica, Escola de Medicina, Universidade Federal de Minas Gerais, Belo Horizonte, Minas Gerais, Brazil; 3 Ambulatório de Dermatologia, Hospital das Clínicas, Universidade Federal de Minas Gerais, Belo Horizonte, Minas Gerais, Brazil; Njala University, SIERRA LEONE

## Abstract

**Objective:**

To identify the prevalence of people with leg ulcers resulting from sickle cell disease, as well as to describe the clinical, social, economic, and demographic conditions of these people.

**Method:**

Descriptive study, carried out at the Minas Gerais Hematology and Hemotherapy Center Foundation. The study population consisted of individuals over the age of 18 with a diagnosis of sickle cell disease in the State of Minas Gerais, Brazil. Data collection was performed from August 2019 to April 2020 through interviews. For the prevalence calculation, a census was taken of 5,379 people over the age of 18 with sickle cell disease, 77 of whom had active leg ulcers. Descriptive data analysis was performed using SPSS software (version 20.0, Chicago, IL, USA).

**Results:**

The prevalence of people with leg ulcers in Minas Gerais, Brazil was 1.4%. Of the 72 respondents, the average age was 39 years (range 18–64 years), 41.7% were single, 48.6% said they were black, 84.7% lived in their own house, 38.9% were retired, 61.1% had an income of one minimum wage. The median years of education was 10.5, 50% cited the church as a place for leisure activities, 79.2% denied smoking. Regarding pain, the median score was 3, the median baseline hemoglobin was 7.7 g/dL, and 91.7% had the HbSS genotype. The median age of the first ulcer was 18 years, 77.8% of active ulcers were recurrent, and 59.7% had only one active ulcer. The median time of existence of the ulcer was 3 years. The ulcer prevented 80.6% of people from doing some activity. Prejudice was experienced by 48.6% of the participants.

**Conclusion:**

The estimated prevalence of leg ulcers was lower than what it reported in the literature, however, the recurrence and the duration of ulcers were high. These findings bring reflection about the assistance to people with leg ulcer.

## Introduction

Sickle cell disease (SCD) comprises a group of inherited diseases resulting from a mutation in the gene that produces hemoglobin A (HbA), leading to the production of hemoglobin S (HbS). The HbS can be inherited in homozygous and heterozygous states, the former being called hemoglobin SS (HbSS), also known as sickle cell anemia. The heterozygous state, on the other hand, caused by inheriting HbS in combination with another mutation in the beta-globin gene, gives rise to hemoglobin Sβ^0^-thalassemia (HbSβ^0^-thal), hemoglobin Sβ^+^-thalassemia (HbSβ^+^thal), and hemoglobin SC (HbSC). HbSS and HbSβ^0^-thal are clinically similar; these genotypes are associated with more severe clinical manifestations, among them leg ulcers [1].

Leg ulcers mainly affect young people, may appear after trauma or spontaneously, often occur on the malleoli, and may involve the entire circumference of the leg. They can have a slough-covered wound bed as well as well-defined and slightly elevated edges. In addition, they are extremely painful and difficult to heal, often lasting months to years with high recurrence rates, reaching 77.8% [1–4].

The pathogenesis of leg ulcers is complex. The exact cause remains unclear, but there is consensus that it is multifactorial. Mechanical obstruction of the microcirculation, hemolysis-vascular dysfunction syndrome, venous incompetence, hypercoagulability and thrombosis, autonomic dysfunction, bacterial colonization, and genetic factors have been described as contributing factors [5]. Ulcers are more common in the second decade of life and in males and are associated with lower socioeconomic status and low education [6]. The prevalence and incidence of leg ulcers vary geographically, according to age and type of SCD [2]. A study in Nigeria identified a 0.45% incidence and 3.1% prevalence of leg ulcers [7]; another study in the United States showed a 2.5% prevalence of leg ulcers and a 5.73% incidence per year in people with SSα-thalassemia and 9.97% in people with HbSS alone [8]. By contrast, in Saudi Arabia, an average 10-year follow-up of people with sickle cell disease recorded no cases of leg ulcers [9].

In Brazil, mainly in Minas Gerais, the prevalence of leg ulcers is unknown. Besides the scarcity of publications that address social, economic, demographic issues and clinical characteristics of these people, when found, they do not directly address these issues and do not cover the entire country. This hinders the implementation of public policies aimed at assistance.

Given the above, this study aimed to identify the prevalence of leg ulcers in individuals with SCD and describe the associated clinical, social, economic, and demographic conditions. The knowledge generated will enable health care providers to improve medical care for these patients.

## Materials and methods

### Study design

Observational, descriptive study.

### Study scenario

The study was carried out at the Minas Gerais Hematology and Hemotherapy Center Foundation (Hemominas). Hemominas Foundation has 11 units in 11 cities in Minas Gerais, Brazil, assisting people with SCD. All people with SCD in Minas Gerais are registered with this foundation.

### Study population and sample

The study population consisted of 5,379 people over 18 years old, diagnosed with SCD and registered at the 11 units previously mentioned.

The diagnosis of sickle cell disease was done at birth using the Newborn Heel Stick Test for the participants who were19 years old, since this test was only available in Brazil in 2001. Participants who were above19 years old were diagnosed using the hemoglobin electrophoresis test. In order to be registered at the Hemominas Foundation, every patient has to be previously diagnosed with sickle cell disease.

The state census was conducted and 77 people with active leg ulcers were identified, of which 72 were interviewed.

### Study variables

The study variables were sociodemographic: place of birth, age, sex, race/color, marital status, housing, treated water, sewage system, and garbage collection; socioeconomic: professional status, individual monthly income, education categorized as uneducated (0 years of study), incomplete elementary school (≥ 1 year < 8 years), complete elementary school (8 years), incomplete high school (> 8 years < 11 years), complete high school (≥ 11 years), and reason for interrupting studies; clinical: sickle cell disease genotype, alcoholism, smoking, age of first ulcer, presence of ulcer history, number of active ulcers, time of existence of the oldest lesion, recurrence, associated diseases, base hemoglobin [10] (< 5 = indicative of nonhealing, > 5 and < 8 = indicative of healing but not ideal, ≥ 8 and < 10 = acceptable for healing, ≥ 10 and ≤ 20 = ideal for healing, > 20 = indicative of nonhealing). Other variables were leisure activities, what prejudices the person has suffered, and what the person has stopped doing because of the wound.

### Data collection

Data collection took place from August 2019 to April 2020 through structured interviews using the researchers’ form in person. The interviews were held in the facilities of the Hemominas Foundation. The Hemominas Foundation does not have a system to identify people with leg ulcers directly; for this reason, the foundation’s doctors, nurses, psychologists, and social workers were essential for this identification. Besides these professionals, we also counted on the cooperation of associations of people with sickle cell disease in Minas Gerais and on the interviewees themselves.

All data and variables were obtained by interview using a data collection form with social demographic status, medical history and lifestyle habits, and also ulcer history.

### Statistical analysis

To estimate the prevalence of people with leg ulcers in the state of Minas Gerais, the following formula was adopted (Eq 1):

$$Prevalence=\frac{Number\;of\;people\;with\;leg\;ulcers}{Number\;of\;people\;with\;sickle\;cell\;disease}\times 100$$ (1)

Descriptive analysis of the data was performed with the Statistical Package for Social Sciences software (SPSS, version 20.0, Chicago, IL, USA) and expressed as percentages, minimum and maximum values, median, and mean.

### Ethical considerations

The study was conducted following the ethical principles originating from the Declaration of Helsinki. The Research Ethics Committees approved the project of the Hemominas Foundation and the Federal University of Minas Gerais in opinions No. 08052818.3.3001.5118 and 08052818.3.0000.5149, respectively. All study participants signed the free and informed consent form.

## Results

The prevalence of people with leg ulcers in Minas Gerais/Brazil was 1.4%. For the calculation, a population of 5,379 people over the age of 18 with SCD was used as the denominator, of which 77 people with leg ulcers were identified. As noted previously, 72 consented for interview.

Of the 72 study participants, 64 (88.9%) were from Minas Gerais, 30 (41.7%) were single, 35 (48.6%) declared themselves black and 35 (48.6%) brown, the age ranged from 18 to 64 years (mean 39 years), 61 (84.7%) lived in their own house, 64 (88.9%) had treated water, 54 (75%) had sewage system, and 63 (87.5%) had garbage collection (Table 1).

**Table 1:** Sociodemographic variables, by sex (n = 72).

Variables	Male n (%)	Female n (%)	Total n (%)
	35 (48.6)	37 (51.4)	72 (100)
**Birthplace**			
Minas Gerais	30 (41.7)	34 (47.2)	64 (88.9)
Espírito Santo	2 (2.8)	2 (2.8)	4 (5.6)
Bahia	1 (1.4)	1 (1.4)	2 (2.8)
Distrito Federal	1 (1.4)	0 (0.0)	1 (1.4)
São Paulo	1 (1.4)	0 (0.0)	1 (1.4)
**Age group**			
Young adolescent	0 (0.0)	2 (2.8)	2 (2.8)
Young adult	5 (6.9)	3 (4.2)	8 (11.1)
Adult	29 (40.3)	30 (41.7)	59 (81.9)
Elderly	1 (1.4)	2 (2.8)	3(4.2)
Race or color*			
Caucasian	0 (0.0)	1 (1.4)	1 (1.4)
Black	16 (22.2)	19 (26.4)	35 (48.6)
Mulatto	19 (26.4)	16 (22.4)	35 (48.6)
Yellow	0 (0.0)	1 (1.4)	1 (1.4)
**Marital status**			
With partner	21 (29.2)	21 (29.2)	42 (58.3)
Without partner	14 (19.4)	16 (22.2)	30 (41.7)
**Housing**			
Owned/lent	31 (43.1)	30 (41.6)	61 (84.7)
Rented	4 (5.6)	7 (9.7)	11 (15.3)
**Treated water**			
No	5 (6.9)	3 (4.2)	8 (11.1)
Yes	30 (41.7)	34 (47.2)	64 (88.9)
**Sewage system**			
No	8 (11.1)	10 (13.9)	18 (25.0)
Yes	27 (37.5)	27 (37.5)	54 (75.0)
**Garbage collection**			
No	3 (4.2)	6 (8.3)	9 (12.5)
Yes	32 (44.4)	31 (43.1)	63 (87.5)

* Self-declared.

Out of the respondents, 28 (38.9%) were retired, 44 (61.1%) had an income of one minimum wage. The median years of education was 10.5 (quartile 1 = 5.0 and quartile 3 = 11.0), with 36 (50%) having completed high school, 2 (2.8%) having finished college, and 29 (40.4%) reporting the clinical repercussions of the disease as the main reason for interrupting their studies (Table 2).

**Table 2:** Socioeconomic characteristics, by sex (n = 72).

Variables	Male n (%)	Female n (%)	Total n (%)
	35 (48.6)	37 (51.4)	72 (100)
**Professional status**			
Self-employed worker	4 (5.6)	1 (1.4)	5 (6.9)
Formal employee	4 (5.6)	0 (0.0)	4 (5.6)
Unemployed	6 (8.3)	11 (15.3)	17 (23.6)
Beneficiaries of Social Security	5 (6.9)	13 (18.1)	18 (25.0)
Retired/Pensioner	16 (22.2)	12 (16.7)	28 (38.9)
Individual monthly income*			
0	6 (0.3)	11 (15.3)	17 (23.6)
< 1	2 (2.8)	1 (1.4)	3 (4.2)
1	20 (27.8)	24 (33.3)	44 (61.1)
> 1	7 (9.7)	1 (1.4)	8 (11.1)
**Education**			
Uneducated/No formal school	1 (1.4)	1 (1.4)	2 (2.8)
Incomplete elementary school	9 (12.5)	12 (16.7)	21 (29.2)
Complete elementary school	4 (5.6)	2 (2.8)	6 (8.3)
Incomplete high school	4 (5.6)	3 (4.2)	7 (9.7)
Complete high school	17 (23.6)	19 (26.4)	36 (50.0)
**Higher education**			
No	30 (41.7)	33 (45.8)	63 (87.5)
Yes	1 (1.4)	1 (1.4)	2 (2.8)
Incomplete	4 (5.6)	3 (4.2)	7 (9.7)
**Reason for study interruption**			
Financial conditions	6 (8.3)	3 (4.2)	9 (12.5)
Distance from school	3 (4.2)	3 (4.2)	6 (8.3)
Disease-related factors	11 (15.3)	18 (25.1)	29 (40.4)
Personal decision	1 (1.4)	2 (2.8)	3 (4.2)

*Minimum wage (Brazil): R$ 1,045.00 (2020).

As for leisure activities, 36 (50%) cited church as their preferred activity and 30 (41.7%) watching television. Prejudice due to the ulcer was experienced by 35 (48.6%) people, of which 27 (37.5%) experienced embarrassing looks. The presence of the ulcer prevented 58 (80.6%) people from doing anything, with attending social occasions reported by 26 (36.1%) (Table 3).

**Table 3:** Data regarding the variables: Leisure activities, forms of prejudice, and activities not performed because of ulcer, by sex (n = 72).

Variables	Male n (%)	Female n (%)	Total n (%)
	35 (48.6)	37 (51.4)	72 (100)
Leisure activity*			
None	3 (4.2)	5 (6.9)	8 (11.1)
Going to church	20 (27.8)	21 (29.2)	36 (50.0)
Walking	3 (4.2)	3 (4.2)	6 (8.3)
Reading	3 (4.2)	5 (6.9)	8 (11.1)
Browsing the social networks	6 (8.3)	5 (6.9)	11 (15.3)
Watching TV	13 (18.1)	17 (23.6)	30 (41.7)
Going out with friends and family	12 (16.7)	9 (12.5)	21 (29.2)
Others	9 (12.6)	3 (4.2)	12 (16.8)
Experienced prejudices*			
Derogatory nicknames	5 (6.9)	2 (2.8)	7 (9.7)
Manifestation of disgust	4 (5.6)	8 (11.1)	12 (16.7)
Uncomfortable looks	10 (13.9)	17 (23.6)	27 (37.5)
Difficulty in finding work	3 (4.2)	2 (2.8)	5 (6.9)
Restricted access to social environments	3 (4.2)	2 (2.8)	3 (4.2)
Fear of contagion	2 (2.8)	2 (2.8)	4 (5.6)
Exclusion due to ulcer*			
Wearing shoes	7 (9.7)	1 (1.4)	8 (11.1)
Wearing skirts or shorts	2 (2.8)	16 (22.2)	18 (25.0)
Getting into water (shower, sea and river)	10 (13.9)	5 (6.9)	15 (20.8)
Traveling	2 (2.8)	6 (8.3)	8 (11.1)
Playing ball games	7 (9.7)	1 (1.4)	8 (11.1)
Attending social events	9 (12.5)	17 (23.6)	26 (36.1)
Walking with ease	1 (1.4)	2 (2.8)	3 (4.2)
Studying	3 (4.2)	5 (6.9)	8 (11.1)
Working	9 (12.5)	3 (4.2)	12 (16.7)
Dating	2 (2.8)	0 (0.0)	2 (2.8)

*The variation in n is due to the choice of more than one option.

Regarding the clinical variables (Table 4), 66 (91.7%) had the HbSS type of sickle cell disease, 57 (79.2%) denied smoking, 68 (94.4%) had an ulcer in the past that had already healed. The median age of the first ulcer was 18 years (quartile 1 = 15 and quartile 3 = 27), with 41 (56.9%) having their first ulcer between the ages of 10 and 20 years, 56 (77.8%) of the active ulcers were recurrent, and 43 (59.7%) had only one active ulcer. The median length of ulcer existence was 3 years (quartile 1 = 0.53 and quartile 3 = 7.7), with 17 (23.6%) with 6 months or less of existence. Regarding pain, the median ulcer pain score was 3 (quartile 1 = 0.53 and quartile 3 = 7.75), and 29 (40.3%) reported severe pain.

**Table 4:** Clinical variables, by sex (n = 72).

Variables	Male n (%)	Female n (%)	Total n (%)
	35 (48.6)	37 (51.4)	72 (100)
**Sickle cell disease subtype**			
HbSS	33 (45.8)	33 (45.8)	66 (91.7)
HbSβ^0^-thal	1 (1.4)	1 (1.4)	2 (2.8)
HbSC	0 (0.0)	2 (2.8)	2 (2.8)
Did not know	1 (1.4)	1 (1.4)	2 (2.8)
**Continuous-use medication**			
Hydroxyurea	26 (36.1)	27 (37.5)	53 (73.6)
Folic acid	32 (44.4)	36 (50.0)	68 (94.4)
Iron chelator	3 (4.2)	1 (1.4)	4 (5.6)
Zinc sulfate	1 (1.4)	1 (1.4)	2 (2.8)
**Sporadic-use medication**			
Analgesic and antipyretic	32 (44.4)	33 (45.8)	65 (90.3)
Opioid analgesic	20(27.8)	23 (31.9)	43 (59.7)
Nonsteroidal anti-inflammatory	3 (4.2)	5 (6.9)	8 (11.1)
Muscle relaxant	2 (2.8)	1 (1.4)	3 (4.2)
Ulcer pain score (VAS)*			
Painless	7 (9.7)	6 (8.3)	13 (18.1)
Low (≥ 1 to ≤ 3)	5 (6.9)	4 (5.6)	22 (30.6)
Moderate (≥ 4 to ≤ 6)	12 (16.7)	9 (12.5)	21 (29.2)
Intense (≥ 7 to ≤ 10)	11 (15.3)	18 (25.0)	29 (40.3)
**Smoking**			
No	26 (36.1)	31 (43.1)	57 (79.2)
Yes	3 (4.2)	4 (5.6)	7 (9.7)
Abstinence	6 (8.3)	2 (2.8)	8 (11.1)
**Age of the first ulcer**			
≤ 10	1 (1.4)	3 (4.2)	4 (5.6)
> 10 to ≤ 20	21 (29.6)	20 (28.2)	41 (56.9)
> 20 to ≤ 30	9 (12.7)	5 (7.0)	14 (19.4)
> 30 to ≤ 40	2 (2.8)	7 (9.9)	9 (12.5)
> 40 to ≤ 50	1 (1.4)	2 (2.8)	3 (4.2)
**History of previous ulcers**			
No	1 (1.4)	3 (4.2)	4 (5.6)
Yes	34 (47.2)	34 (47.2)	68 (94.4)
**Number of active ulcers**			
1	20 (27.8)	23 (31.9)	43 (59.7)
2	9 (12.5)	10 (13.9)	19 (26.4)
3 to 10	6 (8.4)	4 (5.6)	10 (13.9)
**Time of existence of the ulcer (years)**			
≤ 0.5	10 (13.9)	7 (9.7)	17 (23.6)
> 0,5 to ≤ 2	9 (12.5)	8 (11.1)	16 (22.2)
> 2 to ≤ 5	6 (8.3)	8 (11.1)	14 (19.4)
> 5 to ≤ 10	6 (8.3)	5 (6.9)	11 (15.3)
> 10 to ≤ 45	4 (5.6)	9 (12.5)	13 (18.1)
**Recurrence**			
No	7 (9.7)	9 (12.5)	16 (22.2)
Yes	28 (38.9)	28 (38.9)	56 (77.8)

*Visual analog scale of pain. The highest pain score was considered when more than one ulcer was present.

Out of the study participants, 22 (30.6%) had some kind of disease, and in 15 (20.8%) such disease affected the cardiocirculatory system, in 7 (9.7%) the nervous system, in 7 (9.7%) the endocrine system, in 5 (6.9%) the urinary system, and in 1 (1.4%) the digestive system. Alcoholism was present in 4 (5.6%) and 10 (13.9%) were abstinent. The median baseline hemoglobin was 7.7 g/dL (range 7.0–9.0g/dL).

## Discussion

The prevalence of leg ulcers identified in this study was lower than what has previously been published in other countries and in a small study conducted in Minas Gerais/Brazil, which was 5% [11]. Some hypotheses were established for the divergence of the data found. One reason could be related to the fact that in Brazil there is no obligation to notify leg ulcers, which makes the existence of a national database with this information unfeasible. Another hypothesis could be linked to the study design, which included only participants with active ulcers at the time of data collection to estimate the prevalence. Unlike other studies that have included patients with active ulcers and those who have had them [12]. A multicenter, international study of nearly 700 people with SCD identified a leg ulcer prevalence of 10.8%, with 18.6% in Ghana, 3.5% in Italy, and 2.4% in the United States [12]. In Nigeria, 466 medical records of people with SCD aged 16 years and older were reviewed and identified a 3.1% prevalence of leg ulcers [7], yet in this country, the majority in another study involving 250 people with sickle cell disease was 9.6% [13]. Discussion and comparison of ulcer prevalence are made difficult due to the different methodologies used in each study.

The average age of these people in the present study was 39 (18–64) years, a figure higher than that found in Nigeria, with an average of 28.3 (19–40) years [7], and in the United States, Italy, and Ghana, which average was 29.7 (18.0–73.9) years [12]. Although studies cite that ulcers are more common in men, reaching a ratio of 3:1 [13], 2:1 [6] and 1.3:1 [7], in the present study, the variation between both sexes was slight, with a higher occurrence among women.

The presence of the ulcer has deleterious effects on the person’s life concerning education, employment, recreation, and marital and family life. However, the level of education was identified as centric to many of the social consequences [14]. In Brazil, people with sickle cell disease have lower education than people without the disease (p = 0.020) [15]. However, when comparing people with the disease, with and without the ulcer, those with the ulcer left school at a significantly younger age (14.4 years) than those without the ulcer (16.1) (p < 0.01) and the decision to leave was attributed to the teacher, doctor, parent or guardian [14].

In this investigation, 50% of the people with active ulcers did not complete high school. The decision to leave was attributed to the teacher, doctor, parent or guardian, the same justifications found in Jamaica [14]. To exemplify the reason for dropping out of school, one of the study participants, who was of school age and unable to wear shoes due to a malleolar ulcer, reported that he dropped out of school because the principal would not allow him to go to class wearing slippers. This testimonial, among others, reinforces the need for Primary Health Care professionals to work in schools, particularly with teachers, to address sickle cell disease and the limitations it causes, considering that the ulcer appears at school age. This activity should be developed by the School Health Program [16].

Sickle cell disease is not widely known in educational institutions, even when enrolled students with the diagnosis. This fact limits the school’s involvement and its actors with the problems experienced by the student [17].

This research revealed that 25% of the respondents were away from their work activities due to inability to work and were receiving benefits from the Brazilian National Institute of Social Security (INSS), a figure similar to that found in a study conducted in the United States, in which 30% of the participants were away from work [18]. The number of people with ulcer sidelined is higher than those who have the disease (6.4%) [19]. This shows how debilitating the ulcer is, making the subjects unable to perform their work activities.

Another aspect that represents a negative impact, leading to sadness and social isolation, is the prejudice and discrimination suffered by these patients [20], especially those with leg ulcers. This situation can be the trigger for emotional distress [18].

In a study [20] conducted with 113 patients with SCD, participants had the perception that their quality of life was physically and mentally impaired. Patients who reported a perception of prejudice had a statistically significant worse quality of life, revealing that the negative impact can lead to sadness and social isolation. These data reinforce that the presence of the disease is a deeply worrying factor for most people with SCD, as it deteriorates their self-esteem, leading to the construction and perception of a negative self-image [21].

This condition can be worsened in the presence of the ulcer because its treatment imposes the need for frequent dressing change, with increased pain during the procedure and the presence of bandages on the leg, reasons for leading people to social isolation.

The leisure activities of people with ulcers are limited or replaced by activities that do not require much physical movement [14], as is the case for 41.7% of the people in this study for whom watching television was the main leisure activity. Attending church was also one of the leisure activities reported by half of the participants. In this context, religion is considered a way to ease the psychosocial burden of the disease and the associated leg ulcers [18].

A Brazilian qualitative study, developed to understand the experiences of women with sickle cell disease and leg ulcers, showed that participants experienced intense pain and feelings of shame and worthlessness, translated into suffering, low self-esteem, and limitations in social life, which in turn motivated the behavior of isolation [22].

The deprivation of certain activities because of the ulcer can lead the person to social isolation. In this paper, 36.1% of the participants reported not attending social events and 25% modified their clothing after the ulcer onset. Many have stopped wearing clothes that show off their lower limbs, which occurs most often among women. These attitudes may have been taken to hide the ulcer and dressing to avoid stares, questions, and comments and, consequently, constraint [18, 22].

Embarrassing looks from others were reported as the main form of prejudice experienced, followed by disgust from others. The participants’ statements in other studies confirm the data found in this research; for example: "people are disgusted to come near because of the injury. Because of the illness, and still with the ulcer in my leg, I feel different from other people." [22, p. 2075]; "it’s hard for me to be a woman too. I don’t like to go to the beach or things like that because I always cover my ulcers. I usually always wear socks, even in winter or summer" [18, p. 8].

Health care professionals should strongly consider the emotional/psychological effects of SCD when providing care for patients, particularly in the setting of leg ulcers. The search for and adoption of effective treatments are paramount to mitigate the time of ulcer existence and prevent recurrences. Early healing of the ulcer tends to decrease the person’s exposure time to looks and behaviors lacking empathy and compassion. It is important to remember that the presence of the ulcer triggers changes that go beyond the physical dimension.

Individuals with SCD are at high risk for developing multisystem acute and chronic conditions associated with significant morbidity and mortality. Although treatment of SCD may ameliorate some of these complications, such therapies are often unsuccessful in completely preventing them. In the study sample, 22 participants (30.6%) had some kind of associated disease. Therefore, the next best recommendation may be screening to identify risk factors and early signs of complications in order to implement measures to reduce morbidity and mortality in individuals with SCD [1].

The HbSS genotype is the most common and clinically impactful, and leg ulcers are found more frequently in this subpopulation [3, 4, 7, 8, 12, 13, 23–25], a fact confirmed in this study.

In Brazil, the clinical protocol for treating the disease recommends using hydroxyurea, folic acid, and iron chelator [26]. In the current study, 73.6% of participants with leg ulcers used hydroxyurea. However, there isn’t a specific protocol for the systemic or topical treatment. The hydroxyurea is suspected of causing ulcers in patients with the disease, but such a cutaneous side effect remains controversial [27]. In a systematic review study, the use of hydroxyurea to treat people with sickle cell disease was not significantly associated with an increase in the occurrence of leg ulcers [28].

Chronic sickle cell pain syndromes include leg ulcers [29]. They are painful and often disabling complications. This pain may be severe, excruciating, piercing, throbbing, and pungent in nature, unlike a pain crisis resulting from a falciform crisis [18, 30]. People with sickle cell disease and chronic pain often suffer from psychological problems and comorbidities, such as depression, paranoia, and feelings of hopelessness. Many become so preoccupied with their pain that they gradually withdraw from social activities. Existence can be reduced to going from home to health care facilities and pharmacies [30]. In the present study, 82% of the participants experienced ulcer pain, and 40% rated the pain as severe (7 to 10). A similar score, with a mean of 6.5 (5 to 8), was found in another study of 98 people with leg ulcer [3].

In most patients, oral or parenteral opioids are necessary to relieve the pain [30]. In addition to drug therapy, managing the person with pain requires healthcare professionals to be patient, understanding, and empathetic throughout the treatment [29].

About 90% of people in the current study were using analgesics and antipyretics, whether or not associated with opioids, which may indicate undertreatment of their pain; and 59.7% of people were using opioid painkillers, a lower figure than that found in a study of 40 people with leg ulcers, which was 80% [4].

Most ulcers first appear in people between the ages of 10 and 20 years [24, 31]. This was confirmed by the present study, which found 56.9% of people with their first ulcer in this age group (median age 18 years). This result is also similar to a cohort of 659 people with active ulcers or previous history of ulcers whose mean age of onset of the first ulcer was 17.9 years [12].

Of the respondents, 5.6% had their first ulcer, while the majority had a previous ulcer. Prior history of ulcers varies by country: 10.3% in Ghana, Italy and the United States [12], 23.2% in Jamaica [13] and 83% in France [3], the latter being closest to the data found in this research.

After ulcer healing, recurrence is approximately 6 to 8 months [4, 8] and can occur within 2 months [3]. When ulcer healing occurs, the new tissue has less tensile strength than the original skin and is prone to reopen, particularly with increased leg edema, and local trauma. Thus, people with a history of leg ulcers should be followed routinely by health care professionals to prevent recurrence [2].

In the study, about 77.8% of active ulcers were recurrences, a higher number than in other studies, 22.4% [4] and 72.0% [3]. This vast difference in the number of recurrences may be related to the level of instruction by health professionals and the attention of patients to this instruction and the follow-up network of these people after cure.

Another factor that interferes with ulcer healing is the hemoglobin value, including 49.3% of the participants had this value between 5 and 8 g/dL, an acceptable value for healing, but the ideal is in the range of ≥ 10 to ≤ 20 g/dL [10]. In other studies, in 58.4% of people the hemoglobin value was between 6 and 8 g/dL [13] and with means of 7.2 [4], 7.4 [32] and 7.1 g/dL [25].

As for the number of ulcers, 59.7% of patients had an active ulcer, this finding corroborates with other studies, which predominance of single ulcer ranged from 58–76% of cases [3, 4, 13]. In the present research, the median length of ulcer existence was 3 years, a finding close to a study that identified the median length of duration as 4.6 years [12]. However, 23.6% had an ulcer with a length of existence of 6 months or less, a higher figure than that identified in a study conducted in Nigeria, in which ulcer duration of fewer than 6 months was found in 14% [7].

The duration of the ulcer is a predictive factor for healing. Duration shorter than 9 weeks was significantly (p = 0.024) associated with a higher chance of resolution, with an odds ratio of 3.19 (1.16–8.76) [3]. This data can be considered an assistance indicator to evaluate the nursing care provided to people with ulcers. Professionals need to reflect on the care plan for patients in the various points of the Health Care Network and the identification and rapid and appropriate management of the ulcer with topical dressing and edema management.

## Limitations

Regarding the limitations and difficulties encountered in the development of this study, we highlight the data collection that did not include wound assessment due to the difficulty in obtaining an appropriate office for the process of wound assessment and dressing change. In addition, the participant provided all information in this survey, which may lead to memory bias and impact the results. It is essential to point out that at the Hemominas Foundation there is an electronic medical record system, but it does not allow the selection of patients with leg ulcers. This fact may have impacted the identification of the number of people with leg ulcers and the prevalence may have been underestimated, considering the values from other published studies. A more thorough investigation of other states may help determine the question of incidence and prevalence of leg ulcers in Brazil.

## Conclusions

The prevalence of leg ulcers in people with sickle cell disease in Minas Gerais was lower than that described in the literature. The high recurrence rate and the length of time the ulcer lasts draw attention and make us reflect on the inefficiency of the network of care for people with leg ulcers.

## Supporting information

S1 File (PDF)

S2 File (PDF)

S1 Data (DOCX)

S2 Data (DOCX)
