# Supplementary material for: Prevalence of people with sickle cell disease and leg ulcers in Brazil: Socioeconomic and clinical overview
Source: PLoS One. 2022 Sep 9;17(9):e0274254. doi: 10.1371/journal.pone.0274254 (PMC9462796; doi:10.1371/journal.pone.0274254)
Supplement: S1 File — (PDF) [file pone.0274254.s001.pdf]

# PARECER CONSUBSTANCIADO DO CEP

## DADOS DO PROJETO DE PESQUISA

**Título da Pesquisa:** DOENÇA FALCIFORME: TRAJETOS TERAPÊUTICOS DE PESSOAS COM ÚLCERA DE PERNA NOS SERVIÇOS DE ATENÇÃO À SAÚDE

**Pesquisador:** Eline Lima Borges

**Área Temática:**

**Versão:** 2

**CAAE:** 08052818.3.0000.5149

**Instituição Proponente:** Escola de Enfermagem

**Patrocinador Principal:** Financiamento Próprio

## DADOS DO PARECER

**Número do Parecer:** 3.340.212

**Apresentação do Projeto:**

Trata-se de apreciação de resposta à diligência do projeto de pesquisa de estudo observacional do tipo caso -controle que será realizado no estado de Minas Gerais, por meio da Rede Hemominas. Esse trabalho visa pesquisar o percurso terapêutico realizado pelos mineiros com úlcera de perna decorrente da doença falciforme nos serviços de saúde. Minas Gerais é o terceiro estado em termos de número de pessoas com doença falciforme. Para a composição da amostra serão considerados os pacientes em acompanhamento nos Hemocentros distribuídos nas sete macrorregiões de saúde do território de Minas Gerais que estão no Norte (Montes Claros), Triângulo do Norte (Uberlândia), Triângulo do Sul (Uberaba), Centro (Belo Horizonte), Leste (Governador Valadares), Sudeste (Juiz de Fora) e Sul (Pouso Alegre). As demais macrorregiões que não dispõem de Hemocentro, a amostra incluirá pacientes acompanhados nos Hemonúcleos e contemplará Noroeste (Patos de Minas), Oeste (Divinópolis), Jequitinhonha (Diamantina), Centro Sul (São João Del-Rei), Leste Sul. Paciente com doença falciforme e úlcera de perna cadastrado nos referidos centros será recrutado para compor o grupo caso. Para cada caso serão recrutados dois pacientes com doença falciforme sem úlcera de perna que irão compor o grupo controle, na proporção de 1:2. O possível participante da pesquisa será convidado a participar do estudo por um dos pesquisadores no dia da consulta previamente agendadas, conforme rotina, para o profissional médico, enfermeiro, psicólogo ou assistente social do Centro a qual pertence. Os profissionais do referido Centro informarão os pesquisadores sobre estas consultas, evitando

**Endereço:** Av. Presidente Antônio Carlos,6627 2º Ad Sl 2005
**Bairro:** Unidade Administrativa II **CEP:** 31.270-901
**UF:** MG **Município:** BELO HORIZONTE
**Telefone:** (31)3409-4592 **E-mail:** coep@prpq.ufmg.br

assim, o repasse de dados do paciente e custos desnecessários. Os pesquisadores irão aos centros para o recrutamento dos potenciais participantes o número de vezes necessário, respeitando o agendamento do paciente para consultas com os profissionais do Hemocentro ou Hemonúcleo. Critérios de inclusão: ter diagnóstico de doença falciforme, estar cadastrado na Fundação Hemominas, ter idade superior a 18 anos, capacidade escutar e verbalizar. A coleta de dados a ser realizada pelos
pesquisadores ocorrerá nas dependências físicas do centro, como por exemplo, o consultório ou a sala de curativo, conforme disponibilidade. No primeiro contato com o participante o pesquisador irá explicar sobre os objetivos e operacionalização da coleta de dados antes de convidá-lo a participar da pesquisa. Os dados da pesquisa serão coletados por meio de entrevista estruturada com a utilização do formulário aplicado presencialmente pelos pesquisadores que são enfermeiros. A úlcera será avaliada neste momento. banco de dados será transferido para o Stata 12.0 e será submetido à análise descritiva e analítica. Os resultados referentes às características demográficas, socioeconômicas e clínicas da amostra serão analisados por meio da estatística descritiva (porcentagem, valores mínimos e máximos, mediana, média e desvio-padrão).A investigação da associação da ocorrência de úlcera de perna com as variáveis independentes será explorada por meio de testes estatísticos apropriados.

**Objetivo da Pesquisa:**

Foram descritos:

Objetivo Primário: Avaliar a ocorrência de úlceras de perna em pessoas com doença falciforme e os determinantes de sua ocorrência, bem como o percurso terapêutico utilizado entre indivíduos cadastrados na rede Hemominas.

Objetivos Secundários:

•Identificar os pontos de atenção de atendimento à pessoa com falciforme e aquela com úlcera.

•Identificar o local e o responsável pelo acompanhamento da pessoa com úlcera e o fornecimento dos materiais para o tratamento da mesma.

•Avaliar a associação entre fatores clínicos, sociodemográficos e a ocorrência da úlcera de perna.

•Caracterizar as úlceras quanto ao número, área lesada, tempo de existência, recidiva e tratamento utilizado.

**Avaliação dos Riscos e Benefícios:**

Os pesquisadores descrevem:

O sujeito da pesquisa estará sujeito a possíveis desconfortos durante a entrevista e poderá sentir-se cansado ou aborrecido ao responder questionários; pode sofrer alterações na autoestima

provocadas pela recordação de memórias negativas. Para reduzir os possíveis desconfortos o sujeito da pesquisa poderá optar por não responder a pergunta, além disso, o pesquisador estará disponível para escutar o tempo que for necessário. A troca de curativo será da mesma forma que o participante realiza. Apenas o desenho da ferida será realizado em um papel transparente para
obtenção da medida da mesma. O procedimento não é invasivo, por isto, não deve causar dor.
Benefícios: Em relação aos benefícios, apontamos que o conhecimento gerado dará visibilidade as pessoas com úlceras de perna e a seu trâmite nos serviços de saúde de Minas Gerais, fornecendo assim subsídios para melhorar a assistência a essa população.

**Comentários e Considerações sobre a Pesquisa:**
Projeto com financiamento próprio, exequível, realizado com coparticipação do Hemominas. Encontra-se bem estruturado do ponto de vista metodológico, com objetivos bem definidos. O conhecimento gerado elucidará os fatores relacionados com o surgimento da úlcera e mostrará como está sendo realizado o tratamento das pessoas com úlcera de perna nos serviços de saúde deste Estado, fornecendo dados para subsidiar os gestores e profissionais clínicos para a reorganização dos serviços de
saúde e a alocação assertiva de recursos humanos e financeiros para assistência. Foram realizadas as alterações solicitadas no projeto de pesquisa, cronograma e TCLE. Os pesquisadores optaram por adequar os objetivos da pesquisa e modificar a forma de apresentação do TCLE, que passará a ser presencial.

**Considerações sobre os Termos de apresentação obrigatória:**
Foram apresentados:
- Folha de rosto preenchida e assinada.
- Parecer aprovado da Câmara do Departamento da Enfermagem Básica, EE-UFMG, em 05/12/18.
- Projeto no formato da Plataforma Brasil e detalhado (modificado)
- Cronograma da pesquisa com as devidas adequações
- Orçamento da pesquisa com as devidas adequações
- TCLE com as devidas adequações
- Carta ao CEP

**Conclusões ou Pendências e Lista de Inadequações:**
Após realizadas todas as adequações sugeridas per este Comitê sou, SMJ, pela aprovação do projeto.

**Considerações Finais a critério do CEP:**

Tendo em vista a legislação vigente (Resolução CNS 466/12), o CEP-UFMG recomenda aos Pesquisadores: comunicar toda e qualquer alteração do projeto e do termo de consentimento via emenda na Plataforma Brasil, informar imediatamente qualquer evento adverso ocorrido durante o desenvolvimento da pesquisa (via documental encaminhada em papel), apresentar na forma de notificação relatórios parciais do andamento do mesmo a cada 06 (seis) meses e ao término da pesquisa encaminhar a este Comitê um sumário dos resultados do projeto (relatório final).

**Este parecer foi elaborado baseado nos documentos abaixo relacionados:**

| Tipo Documento | Arquivo | Postagem | Autor | Situação |
|---|---|---|---|---|
| Informações Básicas do Projeto | PB_INFORMAÇÕES_BÁSICAS_DO_PROJETO_1256461.pdf | 05/04/2019 17:09:59 | | Aceito |
| Outros | CartaCEP2.pdf | 05/04/2019 17:09:04 | Eline Lima Borges | Aceito |
| TCLE / Termos de Assentimento / Justificativa de Ausência | 3_TCLE.docx | 05/04/2019 17:08:13 | Eline Lima Borges | Aceito |
| Orçamento | ORCAMENTO2.docx | 05/04/2019 17:07:45 | Eline Lima Borges | Aceito |
| Cronograma | CRONOGRAMA2.docx | 05/04/2019 17:00:01 | Eline Lima Borges | Aceito |
| Projeto Detalhado / Brochura Investigador | Projetodetalhado2.docx | 05/04/2019 16:59:20 | Eline Lima Borges | Aceito |
| TCLE / Termos de Assentimento / Justificativa de Ausência | TCLE2.docx | 07/02/2019 13:05:37 | Eline Lima Borges | Aceito |
| Folha de Rosto | Folha_rosto.pdf | 10/12/2018 13:44:52 | JOSIMARE APARECIDA OTONI SPIRA | Aceito |
| Outros | Parecer_camara_departamental.pdf | 10/12/2018 11:08:17 | JOSIMARE APARECIDA OTONI SPIRA | Aceito |
| Projeto Detalhado / Brochura Investigador | Projetodetalhado.docx | 10/12/2018 11:04:57 | JOSIMARE APARECIDA OTONI SPIRA | Aceito |
| TCLE / Termos de Assentimento / Justificativa de | TCLE.docx | 10/12/2018 10:53:20 | JOSIMARE APARECIDA OTONI SPIRA | Aceito |

| | | | | |
|---|---|---|---|---|
| Ausência | TCLE.docx | 10/12/2018 10:53:20 | JOSIMARE APARECIDA OTONI SPIRA | Aceito |
| Orçamento | Orcamento.docx | 09/12/2018 23:57:03 | JOSIMARE APARECIDA OTONI SPIRA | Aceito |
| Cronograma | Cronograma.docx | 09/12/2018 23:56:47 | JOSIMARE APARECIDA OTONI SPIRA | Aceito |

**Situação do Parecer:**
Aprovado

**Necessita Apreciação da CONEP:**
Não

BELO HORIZONTE, 22 de Maio de 2019

**Assinado por:**
**Eliane Cristina de Freitas Rocha**
**(Coordenador(a))**
