# Supplementary material for: Prevalence of people with sickle cell disease and leg ulcers in Brazil: Socioeconomic and clinical overview
Source: PLoS One. 2022 Sep 9;17(9):e0274254. doi: 10.1371/journal.pone.0274254 (PMC9462796; doi:10.1371/journal.pone.0274254)
Supplement: S2 File — (PDF) [file pone.0274254.s002.pdf]

PARECER CONSUBSTANCIADO DO CEP

Elaborado pela Instituição Coparticipante

DADOS DO PROJETO DE PESQUISA

Título da Pesquisa: DOENÇA FALCIFORME: TRAJETOS TERAPÊUTICOS DE PESSOAS COM ÚLCERA DE PERNA NOS SERVIÇOS DE ATENÇÃO À SAÚDE

Pesquisador: Eline Lima Borges

Área Temática:

Versão: 4

CAAE: 08052818.3.3001.5118

Instituição Proponente: FUND CENTRO HEMATOLOGIA E HEMOTERAPIA DE MINAS GERAIS

Patrocinador Principal: Financiamento Próprio

DADOS DO PARECER

Número do Parecer: 3.528.886

Apresentação do Projeto:

Segundo informado pelos pesquisadores, trata-se de estudo observacional do tipo caso-controle que será realizado no estado de Minas Gerais no intuito de avaliar o percurso terapêutico de pessoas com úlcera de perna decorrente da doença falciforme nas Redes de Atenção à Saúde, bem como os determinantes de ocorrência da úlcera. A população do estudo será composta por pessoas com doença falciforme cadastradas na Fundação Hemominas, sendo considerados para a composição da amostra os pacientes em acompanhamento nos hemocentros e hemonúcleos, que atendam os seguintes critérios de inclusão:ter diagnóstico de doença falciforme, estar cadastrado na Fundação Hemominas, ter idade superior a 18 anos, capacidade escutar e verbalizar.Paciente com doença falciforme e úlcera de perna cadastrado nos referidos centros será recrutado para compor o grupo caso. Para cada caso serão recrutados dois pacientes com doença falciforme sem úlcera de perna que irão compor o grupo controle, na proporção de 1:2. O possível participante da pesquisa será convidado a participar do estudo por um dos pesquisadores no dia da consulta previamente agendadas, conforme rotina, para o profissional médico, enfermeiro, psicólogo ou assistente social do Centro a qual pertence. Os profissionais do referido Centro informarão os pesquisadores sobre estas consultas, evitando assim, o repasse de dados do paciente e custos desnecessários. Os pesquisadores irão aos centros para o recrutamento dos potenciais participantes o
número de vezes necessário, respeitando o agendamento do paciente para consultas com os

Endereço: Alameda Ezequiel Dias. 321
Bairro: Santa Efigênia **CEP:** 30.130-110
UF: MG **Município:** BELO HORIZONTE
Telefone: (31)3768-4689 **Fax:** (31)3768-4600 **E-mail:** cep@hemominas.mg.gov.br

profissionais do Hemocentro ou Hemonúcleo. A coleta de dados a ser realizada pelos pesquisadores ocorrerá nas dependências físicas do centro, como por exemplo, o consultório ou a sala de curativo, conforme disponibilidade. Os dados da pesquisa serão coletados por meio de entrevista estruturada com a utilização do formulário aplicado presencialmente pelos pesquisadores que são enfermeiros. A úlcera será avaliada neste momento. O banco de dados será transferido para o Stata 12.0 e
será submetido à análise descritiva e analítica. Os dados obtidos serão analisados por meio de estatística descritiva e a investigação da associação da ocorrência de úlcera de perna com as variáveis independentes será explorada por meio de testes estatísticos. Ainda segundo os pesquisadores, o conhecimento gerado dará visibilidade às pessoas com úlcera de perna, mostrará os fatores relacionados com o surgimento da úlcera e mostrará como está sendo realizado o tratamento das pessoas com úlcera de perna nos serviços de saúde deste Estado e fornecerá dados para subsidiar os gestores e profissionais clínicos para a reorganização dos serviços de saúde, a alocação assertiva de recursos humanos e financeiros para assistência e, consequentemente, universalizar o cuidado dessa clientela. Após análise do CEP, foi emitido parecer no qual foram levantadas inúmeras pendências a serem esclarecidas. Diante delas, os
pesquisadores apresentaram suas considerações, promovendo alterações no projeto original e no documento PB_Informações Básicas do Projeto. Mais uma vez, submetido à análise do CEP, pendência relativa à garantia de indenização não foi atendida. Novo parecer de pendência foi emitido, e após ciência da argumentação exposta pelo CEP, os pesquisadores realizaram as adequações necessárias.

Objetivo da Pesquisa:

Objetivo Primário:

Avaliar o percurso terapêutico de pessoas com úlcera de perna decorrente da doença falciforme nas Redes de Atenção à Saúde, bem como os determinantes de ocorrência da úlcera.

Objetivo Secundário:

•Identificar os pontos de atenção de atendimento à pessoa com doença falciforme e aquela com úlcera.

•Identificar o local e o responsável pelo acompanhamento da pessoa com úlcera e o fornecimento dos materiais para o tratamento da mesma.

•Avaliar a associação entre fatores clínicos, sociodemográficos e a ocorrência da úlcera de perna.

•Caracterizar as úlceras quanto ao número, área lesada, tempo de existência, recidiva e

tratamento utilizado.

Avaliação dos Riscos e Benefícios:

Riscos: O participante da pesquisa estará sujeito a possíveis desconfortos durante a entrevista e poderá sentir-se cansado ou aborrecido ao responder questionários; pode sofrer alterações na autoestima provocadas pela recordação de memórias negativas. Para reduzir os possíveis desconfortos o sujeito da pesquisa poderá optar por não responder a pergunta, além disso, o pesquisador estará disponível para escutar o tempo que for necessário. A troca de curativo será da mesma forma que o participante realiza. Apenas o desenho da ferida será realizado em um papel transparente para obtenção da medida da mesma. O procedimento não é invasivo, por isto, não deve causar dor.

Benefícios: Em relação aos benefícios, apontamos que o conhecimento gerado dará visibilidade as pessoas com úlceras de perna e a seu trâmite nos serviços de saúde de Minas Gerais, fornecendo assim subsídios para melhorar a assistência a essa população.

Comentários e Considerações sobre a Pesquisa:

Conforme informado no projeto de pesquisa,"Foram realizadas buscas nos bancos e nas bases de dados; entretanto, não foram localizados trabalhos tratando da trajetória da pessoa de úlcera de perna na rede de atenção à saúde". Os trabalhos existentes sobre a temática abordam questões distintas não relacionadas ao objeto a ser estudado. Sendo assim,a pesquisa proposta apresenta relevância científica por ser capaz de contribuir para a verificação dos fatores relacionados com o surgimento da úlcera e ao tratamento das pessoas com úlcera de perna nos serviços de saúde do Estado. Como dito, após análise do CEP, foram emitidos pareceres nos quais foram levantadas inúmeras pendências a serem esclarecidas. Diante delas, os pesquisadores apresentaram suas considerações, promovendo alterações no projeto original, no documento PB_Informações Básicas do Projeto e no TCLE.

Considerações sobre os Termos de apresentação obrigatória:

Verificar campo "Conclusões ou Pendências e Lista de Inadequações".

Recomendações:

No documento "6_TCLE.docx" consta os nomes da secretária e da coordenadora do CEP-Hemominas. Solicita-se que os nomes sejam suprimidos, mantendo apenas o e-mail para contato com o CEP.

Endereço: Alameda Ezequiel Dias. 321
Bairro: Santa Efigênia **CEP:** 30.130-110
UF: MG **Município:** BELO HORIZONTE
Telefone: (31)3768-4689 **Fax:** (31)3768-4600 **E-mail:** cep@hemominas.mg.gov.br

Conclusões ou Pendências e Lista de Inadequações:

1. O cronograma aponta que a alimentação do banco de dados seria iniciada em 15/05/2019. Entretanto, o projeto ainda está sob apreciação do CEP Hemominas, que não analisa pesquisas já iniciadas. Sendo assim, solicita-se a adequação do crononograma.

RESPOSTA: No projeto, a data de início foi alterada para 4º bimestre de 2019, considerando que o projeto pode ser aprovado na próxima reunião do CEP prevista para ocorrer em julho de 2019. Não foi possível alterar o cronograma da Plataforma.

ANÁLISE: PENDÊNCIA ATENDIDA.

2. A folha de rosto não indica a Fundação Hemominas como instituição co-participante. Solicita-se adequação.

RESPOSTA: Feito impressão de nova folha de rosto onde consta a Fundação Hemominas como instituição proponente, e não como coparticipante. A referida folha foi assinada pelo pesquisador e anexada nos documentos da Plataforma.

ANÁLISE: PENDÊNCIA ATENDIDA.

3. O projeto não menciona o risco de quebra de confidencialidade inerente a todo projeto de pesquisa. Solicita-se, portanto, sua adequação de forma que atenda ao disposto na alínea i, do item III.2 da Resolução CNS 466/12: "i) prever procedimentos que assegurem a confidencialidade e a privacidade, a proteção da imagem e a não estigmatização dos participantes da pesquisa, garantindo a não utilização das informações em prejuízo das pessoas e/ou das comunidades, inclusive em termos de autoestima, de prestígio e/ou de aspectos econômico-financeiros".

RESPOSTA: Incluído informações referentes ao tema no projeto e no TCLE. As informações foram destacadas em vermelho.

ANÁLISE: PENDÊNCIA ATENDIDA. Foi incluído o seguinte trecho: "Visando reduzir o risco de quebra de confidencialidade e garantindo a manutenção do sigilo e da privacidade dos participantes da pesquisa em todas as suas fases, esclarecemos que apenas os pesquisadores terão acesso às informações prestadas e que os dados serão mantidos em local trancado até o momento da publicação dos mesmos. O anonimato será garantido em todas as fases da pesquisa, incluindo a publicação. Será garantida a priv cidade a não estigmatização dos participantes da pesquisa, com proteção da imagem e não utilização das informações em prejuízo das pessoas e/ou das comunidades, inclusive em termos de autoestima, de prestígio e/ou de aspectos econômico-financeiros, uma vez que o participante não será identificado em todas as fases da pesquisa,

incluindo a divulgação dos dados por meio de artigos científicos ou apresentação em fóruns acadêmicos".

4. Solicita-se a indicação da forma de divulgação dos resultados, em especial, para o participante da pesquisa e informar ainda os encaminhamentos a serem adotados no caso de eventuais intercorrências, como por exemplo a constatação de problemas no curativo ou úlcera infectada.
RESPOSTA: Incluído informações no projeto e no TCLE destacadas em vermelho.
ANÁLISE: PENDÊNCIA ATENDIDA. Foi incluído o seguinte trecho: Os dados serão divulgados por meio de artigos científicos ou apresentação em fóruns acadêmicos independentes dos resultados. Os dados também serão compartilhados com a Fundação Hemominas, por meio do envio do relatório final, o qual será de acesso aos profissionais do Hemocentros/Hemonúcleos.

5. Em atendimento ao disposto na alínea i, do item III.2, citado anteriormente, solicita-se a adequação do TCLE de forma que mencione a existência do risco de quebra de sigilo.
RESPOSTA: Para atender as recomendações nas letras e) e f) foi inserido no TCLE: "Visando reduzir o risco de quebra de confidencialidade e garantindo a manutenção do sigilo e da privacidade dos participantes da pesquisa em todas as suas fases, esclarecemos que apenas os pesquisadores terão acesso às informações prestadas e que os dados serão mantidos em local trancado até o momento da publicação dos mesmos. Esclarecemos também que você não será identificado por meio dos dados publicados. O seu anonimato será garantido em todas as fases da pesquisa, incluindo a publicação."
Conclusão: PENDÊNCIA ATENDIDA.

6. Com base no item IV.3, alíneas b e e, da Resolução CNS 466/12, solicita-se a adequação do TCLE de modo que sejam apresentadas as providências e cautelas a serem empregadas para reduzir o risco de quebra de confidencialidade, garantindo a manutenção do sigilo e da privacidade dos participantes da pesquisa em todas as suas fases.
RESPOSTA: Para atender as recomendações nas letras e) e f) foi inserido no TCLE: (...)
ANÁLISE: PENDÊNCIA ATENDIDA.

7. Com fulcro no item IV.3, g, da Resolução CNS 466/12, caso o participante da pesquisa tenha que comparecer ao Hemocentro ou ao hemonúcleo exclusivamente para participar da pesquisa, ou

Endereço: Alameda Ezequiel Dias. 321
Bairro: Santa Efigênia **CEP:** 30.130-110
UF: MG **Município:** BELO HORIZONTE
Telefone: (31)3768-4689 **Fax:** (31)3768-4600 **E-mail:** cep@hemominas.mg.gov.br

permaneça, por tempo prolongado na unidade, em função do estudo, solicita-se a explicitação da garantia de ressarcimento e como serão cobertas as despesas a eles impostas.
RESPOSTA: Foi esclarecido que: Os pesquisadores irão aproveitar o momento de sua consulta com os profissionais do hemocentro ou hemonúcleo, agendada previamente, para a coleta de dados. Entretanto, isto não aumentará em demasia o seu tempo de permanência na unidade, bem como despesas extras relacionadas à pesquisa. A troca de curativo será da mesma forma que você já faz e não implicará gasto financeiro para você.
ANÁLISE: PENDÊNCIA ATENDIDA.

8. Em atendimento ao disposto no item IV.3, h, solicita-se que o TCLE explicite a garantia de indenização diante de eventuais danos decorrentes da pesquisa.
RESPOSTA: Foi incluído no TCLE o seguinte trecho: "Informamos que você poderá apresentar possíveis desconfortos durante a entrevista e poderá sentir-se cansado ou aborrecido ao responder o questionário. Você também pode sofrer alterações na autoestima provocadas pela recordação de memórias negativas. Para reduzir os possíveis desconfortos, você poderá optar por não responder a pergunta, além disso, o pesquisador estará disponível para escutar o tempo que for necessário. Caso o risco diretamente relacionado à pesquisa se concretize, lhe será assegurado a avaliação por profissional competente caracterizando a garantia de indenização diante de eventuais danos decorrentes da pesquisa".
ANÁLISE: PENDÊNCIA NÃO ATENDIDA. Conforme exigência contida no item IV.3, h,da Resolução CNS 466/12,é necessário que os pesquisadores mencionem expressamente que aos participantes será garantida indenização diante de eventuais danos decorrentes da pesquisa, não limitando-a a avaliação por profissionais.
RESPOSTA: Foram incluídas informações no TCLE referentes à garantida de indenização. As alterações foram destacadas em vermelho e constam de: -A troca de curativo será realizada pelo enfermeiro pesquisador utilizando técnica asséptica (o profissional utilizará máscara e luvas de procedimento e estéril, solução fisiológica estéril e gaze estéril) garantindo assim, que você não desenvolva infecção na ferida.
Também será respeitado o tratamento tópico que você já utiliza. (...) Caso a ferida apresente infecção no período de 15 dias decorrente do procedimento realizado para a coleta de dados da pesquisa, lhe será assegurando assistência clínica caracterizando a garantia de indenização.
ANÁLISE: PENDÊNCIA NÃO ATENDIDA. A Resolução CNS 466/12 menciona a garantia de indenização diante de eventuais danos decorrentes da pesquisa. Note-se que a norma fala em

Endereço: Alameda Ezequiel Dias. 321
Bairro: Santa Efigênia **CEP:** 30.130-110
UF: MG **Município:** BELO HORIZONTE
Telefone: (31)3768-4689 **Fax:** (31)3768-4600 **E-mail:** cep@hemominas.mg.gov.br

danos decorrentes da pesquisa, já os pesquisadores a limitam a apenas um tipo de evento adverso, desafiando adequação. Quanto ao tipo de indenização, os pesquisadores informam que se constituirá pela assistência clínica. Ocorre que a garantia de assistência clínica estaria contida no conceito de assistência ao participante da pesquisa, previsto no item II.3, nos subitens II.3.1 e II.3.2, e assegurada no item III.2, "o", nos seguintes termos: "o) assegurar aos participantes da pesquisa as condições de acompanhamento, tratamento, assistência integral e orientação, conforme o caso, enquanto necessário, inclusive nas pesquisas de rastreamento". Também o item V.6 menciona a garantia de assistência, da seguinte forma: "V.6 - O pesquisador, o patrocinador e as instituições e/ou organizações envolvidas nas diferentes fases da pesquisa devem proporcionar assistência imediata, nos termos do item II.3, bem como responsabilizarem-se pela assistência integral aos participantes da pesquisa no que se refere às complicações e danos decorrentes da pesquisa." Já a garantia de indenização consistiria, consoante item II.7, da Resolução, na "cobertura material para reparação a dano, causado pela pesquisa ao participante da pesquisa". Sendo assim, como a garantia de assistência e a garantia de indenização seriam coisas distintas, recomenda-se que os pesquisadores explicitem que aos participantes será garantida indenização diante de eventuais danos decorrentes da pesquisa.
ANÁLISE: PENDÊNCIA ATENDIDA. Em resposta, os pesquisadores incluíram no TCLE a seguinte assertiva: “Esclarecemos que será garantida indenização diante de eventuais danos decorrentes da pesquisa, em respeito à exigência contida no item IV.3, h,da Resolução 466/12 do Conselho Nacional de Saúde (CNS) de 12 de dezembro de 2012. Destacamos que este documento foi elaborado de acordo com a referida Resolução”.

9. Em atendimento ao item IV.5, d, da Resolução CNS 466/12, solicita-se a inclusão do contato do CEP Hemominas.
RESPOSTA: Os dados foram inseridos no TCLE.
ANÁLISE: PENDÊNCIA ATENDIDA.

10. O Item IV.3, a, da Resolução nº466/12 determina que o TCLE obrigatoriamente contenha informação concernente à possibilidade de inclusão do participante em grupo controle. Por esse motivo, e considerando que os participantes da pesquisa serão divididos em grupo caso e grupo controle, mister se faz a elaboração de termos específicos para cada um dos grupos nos quais sejam explicitadas as razões para a inclusão dos participantes em cada um deles ou adequação do termo único para constar a possibilidade do participante ser enquadrado em grupo controle ou

estudo de caso.
RESPOSTA: Feito esclarecimento no texto:"Serão inseridas nesse estudo pessoas com diagnóstico de doença falciforme que residem em Minas Gerais, maiores de 18 anos e cadastrados na Fundação Hemominas. Para os pacientes que não apresentarem úlcera irão pertencer ao grupo controle e responderão somente a entrevista e para aqueles com úlcera na perna, pertencerão ao grupo caso, responderão as perguntas e terão a úlcera mensurada e também responderão perguntas específicas a respeito da úlcera e seu tratamento."
ANÁLISE: PENDÊNCIA ATENDIDA.

11. Importante adequar a forma como será dado o repasse das datas de consultas para os pesquisadores. O repasse dos dados pela Fundação Hemominas deverá ser feito mediante a informação geral do agendamento de consultas, sem qualquer seleção ou identificação de dados do paciente.
RESPOSTA: Foi inserido no documento a seguinte parte: Esclarecemos que não recebemos informações sobre os seus dados pelos profissionais da Fundação Hemominas. Tivemos apenas acesso à informação geral do agendamento de consultas, sem qualquer seleção ou identificação de dados dos pacientes.
ANÁLISE: PENDÊNCIA ATENDIDA.

Considerações Finais a critério do CEP:
Prezado(a) pesquisador(a), seu projeto de pesquisa foi aprovado pelo CEP-Hemominas e, conforme definido pela Resolução CNS 466/12, deve ser acompanhado por meio de relatórios parciais e final. Solicitamos que relatórios parciais sejam apresentados a esse CEP a cada 12 meses a contar a partir da data de aprovação do projeto na Plataforma Brasil. O relatório final deve ser apresentado assim que a pesquisa for encerrada. Os relatórios devem seguir o padrão definido pelo Serviço de Pesquisa da Fundação Hemominas e o formulário a ser utilizado deve ser solicitado pelo e-mail secretaria.pesquisa@hemominas.mg.gov.br. Os relatórios devem ser preenchidos, assinados, digitalizados e submetidos na Plataforma Brasil como "Notificação" para serem analisados pelo CEP-Hemominas. Os(As) pesquisadores(as) que não submeterem seus relatórios serão considerados(as) inadimplentes.

Este parecer foi elaborado baseado nos documentos abaixo relacionados:

Endereço: Alameda Ezequiel Dias. 321
Bairro: Santa Efigênia **CEP:** 30.130-110
UF: MG **Município:** BELO HORIZONTE
Telefone: (31)3768-4689 **Fax:** (31)3768-4600 **E-mail:** cep@hemominas.mg.gov.br

Tipo Documento	Arquivo	Postagem	Autor	Situação
Informações Básicas do Projeto	PB_INFORMAÇÕES_BÁSICAS_DO_PROJETO_1362578.pdf	07/08/2019 09:57:53		Aceito
Outros	OFICIO_2019_agosto.pdf	07/08/2019 09:57:15	Eline Lima Borges	Aceito
TCLE / Termos de Assentimento / Justificativa de Ausência	6_TCLE.docx	07/08/2019 09:53:19	Eline Lima Borges	Aceito
Projeto Detalhado / Brochura Investigador	Projetodetalhado5.docx	07/08/2019 09:53:05	Eline Lima Borges	Aceito
Outros	2OFICIO_PARA_CEP_Hemonimas_2019.pdf	09/07/2019 18:49:11	Eline Lima Borges	Aceito
Projeto Detalhado / Brochura Investigador	Projetodetalhado4.docx	09/07/2019 18:48:04	Eline Lima Borges	Aceito
TCLE / Termos de Assentimento / Justificativa de Ausência	5_TCLE.docx	09/07/2019 18:03:49	Eline Lima Borges	Aceito
Outros	Cadastro_Pesquisa_Assinado.pdf	28/06/2019 11:33:03	Eline Lima Borges	Aceito
Folha de Rosto	Folha_Rosto_Hemominas.pdf	28/06/2019 11:31:25	Eline Lima Borges	Aceito
Outros	OFICIO_PARA_CEP_Hemonimas_2019.pdf	17/06/2019 17:12:45	Eline Lima Borges	Aceito
TCLE / Termos de Assentimento / Justificativa de Ausência	4_TCLE.docx	17/06/2019 17:10:27	Eline Lima Borges	Aceito
Projeto Detalhado / Brochura Investigador	Projetodetalhado3.docx	17/06/2019 17:10:05	Eline Lima Borges	Aceito
Cronograma	CRONOGRAMA3.docx	17/06/2019 17:09:28	Eline Lima Borges	Aceito
Outros	12_03_18_compromisso_externos_josimare.pdf	22/05/2019 18:54:08	Eline Lima Borges	Aceito
Outros	12_03_18_compromisso_externos_eline.pdf	22/05/2019 18:53:06	Eline Lima Borges	Aceito
Outros	12_03_18_termo_compromisso.pdf	22/05/2019 18:51:58	Eline Lima Borges	Aceito
Outros	12_03_18_cadastro_pesquisa.pdf	22/05/2019 18:50:40	Eline Lima Borges	Aceito
Outros	CartaCEP2.pdf	05/04/2019 17:09:04	Eline Lima Borges	Aceito
TCLE / Termos de	3_TCLE.docx	05/04/2019	Eline Lima Borges	Aceito

Assentimento / Justificativa de Ausência	3_TCLE.docx	17:08:13	Eline Lima Borges	Aceito
Projeto Detalhado / Brochura Investigador	Projetodetalhado2.docx	05/04/2019 16:59:20	Eline Lima Borges	Aceito
TCLE / Termos de Assentimento / Justificativa de Ausência	TCLE2.docx	07/02/2019 13:05:37	Eline Lima Borges	Aceito
Outros	Parecer_camara_departamental.pdf	10/12/2018 11:08:17	JOSIMARE APARECIDA OTONI SPIRA	Aceito
Projeto Detalhado / Brochura Investigador	Projetodetalhado.docx	10/12/2018 11:04:57	JOSIMARE APARECIDA OTONI SPIRA	Aceito
TCLE / Termos de Assentimento / Justificativa de Ausência	TCLE.docx	10/12/2018 10:53:20	JOSIMARE APARECIDA OTONI SPIRA	Aceito

Situação do Parecer:
Aprovado

Necessita Apreciação da CONEP:
Não

BELO HORIZONTE, 23 de Agosto de 2019

Assinado por:
Daniel Gonçalves Chaves
(Coordenador(a))
