# Supplementary material for: Prevalence of people with sickle cell disease and leg ulcers in Brazil: Socioeconomic and clinical overview
Source: PLoS One. 2022 Sep 9;17(9):e0274254. doi: 10.1371/journal.pone.0274254 (PMC9462796; doi:10.1371/journal.pone.0274254)
Supplement: S1 Data — (DOCX) [file pone.0274254.s003.docx]

| **DATA COLLECTION INSTRUMENT** | | |
| --- | --- | --- |
| **Questionnaire number:** | **Collection date** ____/_____/____ | |
| **Registered Blood Center** _______________________________________ | | |
| **IDENTIFICATION, SOCIAL ECONOMIC DEMOGRAPHIC STATUS** | | |
| **Birth date** ____/_____/____ | | **Gender:** ( )male ( )female |
| **Birthplace:** | | |
| **Race/ ethnicity** _(self-identified)_**:** ( )Caucasian ( )Non-Caucasian | | |
| **Marital status:** ( ) with partner ( ) without partner | | |
| **Education:** ( ) uneducated/ no formal school ( ) incomplete elementary school ( ) complete elementary school ( ) Incomplete high school ( ) complete high school  **Reason for study interruption:** _________________________ | | |
| **Higher education:** ( ) no ( ) yes ( ) Incomplete | | |
| **Professional Status:** ( )self-employed worker ( )formal employee ( )unemployed ( ) beneficiaries of Social Security( ) Retired/Pensioner | | |
| **Individual monthly income *:** ( ) without income ( )< US$203,70 ( )= US$203,70  ( )> US$203,70 e ≤US$407,40 ( )> US$407,40 e ≤ US$611,10 ( ) US$611,10 | | |
| **Housing:** ( )owned/lent ( )rented  **Treated water**: ( )yes ( )no  **Garbage collection:** ( )yes ( )no  **Sewage system:** ( )yes ( )no | | |
| **MEDICAL HISTORY AND LIFESTYLE HABITS** | | |
| **Smoking:** ( )yes ( )no ( )abstinence | | |
| **Sickle cell disease subtype:** _____________________________________________ | | |
| **Previous ulcer occurrence:** ( )yes ( )no **If yes, specify the age when the first ulcer occurred:**____________ | | |
| **Medication list:** _________________________________________________  _____________________________________________________________________________ | | |
| **Medications taken PRN:** _______________________________________________  _____________________________________________________________________________ | | |
| **Associated diseases:** ___________________________________________________________ ___________________________________________________________________________________________________________ | | |
| **Leisure activities:** ( ) Attending religious services ( )walking ( )practicing sports ( )reading  ( )going to the movies ( ) traveling  ( ) watching TV ( )fishing ( ) going out with friends and family ( ) Others__________________ | | |
| **Presence of active ulcer**: ( )yes ( )no, **If yes, please continue to answer.** | | |
| **Number of active ulcers**: ______ **Recurrence of present ulcer**: ( )yes ( )no | | |
| **Lenth of existence of the oldest ulcer** _(months)_: ______________ | | |
| **Pain score rating of ulcer(s)** ^‡^_(score 0 a 1 0)_: ______ | | |
| **Have you ever faced prejudice because of your wound?** ( )yes ( )no  **If yes, explain**. ______________________________________ | | |
| **Did your leg ulcer ever prevent you from performing activities?** ( )yes ( )no  **If yes, which one affected you the most**?______________________________________ | | |
| Foot note  *****Minimum wage: US$203,70  ^‡^ The Pain Visual Analogue Scale (VAS).   \|  \| \| --- \| | | |

Location, date e data collector’s signature
