# Supplementary material for: Prevalence of people with sickle cell disease and leg ulcers in Brazil: Socioeconomic and clinical overview
Source: PLoS One. 2022 Sep 9;17(9):e0274254. doi: 10.1371/journal.pone.0274254 (PMC9462796; doi:10.1371/journal.pone.0274254)
Supplement: S2 Data — (DOCX) [file pone.0274254.s004.docx]

| **INSTRUMENTO DE COLETA DE DADOS DA PESQUISA** | | |
| --- | --- | --- |
| **Número do questionário:** | **Data da coleta** ____/_____/____ | |
| **Hemocentro/Hemonúcleo cadastrado:** _______________________________________ | | |
| **IDENTIFICAÇÃO, PERFIL SÓCIO- DEMOGRÁFICO E ECONÔMICO** | | |
| **Data de nascimento** ____/_____/____ | | **Sexo:** ( )masculino ( )feminino |
| **Naturalidade:** | | |
| **Raça/cor** _(autodeclarada)_**:** ( )Caucasiano ( )Não caucasiano | | |
| **Estado civil:** ( )Com parceiro ( )Sem parceiro | | |
| **Anos de estudo completos:** ______  **Educação:** ( ) sem instrução/ educação informal ( ) Ensino fundamental incompleto ( ) Ensino fundamental completo ( ) Ensino médio incompleto ( ) Ensino médio completo  **Se interrompeu os estudos, por quê?**______________________________________________  _____________________________________________________________________________ | | |
| **Curso superior:** ( ) não ( ) incompleto ( ) completo | | |
| **Status profissional:** ( )estudante ( )trabalhador autônomo ( )empregado formal  ( )desempregado ( )pensionista ( )aposentado ( ) benefício do Instituto Nacional do Seguro Social | | |
| **Renda individual mensal*:** ( ) sem renda ( )< R$1.045,00 ( )= R$1.045,00 ( )> R$1.045,00 e ≤2.090,00 ( )>2.090,00 e ≤3.135,00 ( )>33.135,00 | | |
| **Habitação:** ( )própria ( )cedida ( )alugada  **Água tratada**: ( )sim ( )não  **Coleta de lixo:** ( )sim ( )não  **Esgoto:** ( )sim ( )não | | |
| **HISTÓRIA PREGRESSA E HÁBITOS DE VIDA** | | |
| **Tabagismo:** ( )sim ( )não ( )abstinência | | |
| **Subtipo de doença falciforme:** _____________________________________________ | | |
| **Ocorrência de úlcera anterior:** ( )sim ( )não **Se sim, idade que surgiu a primeira úlcera:**____________ | | |
| **Medicamentos em uso contínuo:** _________________________________________________  _____________________________________________________________________________ | | |
| **Medicamentos de uso esporádico:** _______________________________________________  _____________________________________________________________________________ | | |
| **Doenças associadas:** ___________________________________________________________ ___________________________________________________________________________________________________________ | | |
| **Atividade de lazer:**( )igreja ( )caminhar ( )praticar esportes ( )leitura ( )cinema ( )viajar  ( )assistir TV ( )pescar ( )sair com os amigos e familiares ( )Outras________________________ | | |
| **Presença de úlcera ativa**: ( )sim ( )não, **Se sim, dê continuidade ao questionário.** | | |
| **Número de lesões ativas**: ______ **Recidiva da(s) úlcera(s) atual**: ( )sim ( )não | | |
| **Tempo de existência da úlcera ativa mais antiga** _(meses)_: ______________ | | |
| **Escore de dor na(s) úlcera(s)** ^‡^_(escore 0 a 1 0)_: ______ | | |
| **Você já sofreu preconceito por causa da ferida?** ( )sim ( )não  **Se sim, qual foi a que mais te marcou**?______________________________________ | | |
| **Você já deixou de fazer algo por causa da ferida?** ( )sim ( )não  **Se sim, qual foi a que mais te marcou**?______________________________________ | | |
| Legenda  *****Salário mínimo: R$1.045,00  ^‡^ Escala visual analógica (EVA) – escore de 0 a 10.   \|  \| \| --- \| | | |

Local, data e Assinatura do responsável pela coleta
